# Transplantation of bioengineered rat lungs recellularized with endothelial and adipose-derived stromal cells

**DOI:** 10.1038/s41598-017-09115-2

**Published:** 2017-08-16

**Authors:** Ryoichiro Doi, Tomoshi Tsuchiya, Norisato Mitsutake, Satoshi Nishimura, Mutsumi Matsuu-Matsuyama, Yuka Nakazawa, Tomoo Ogi, Sadanori Akita, Hiroshi Yukawa, Yoshinobu Baba, Naoya Yamasaki, Keitaro Matsumoto, Takuro Miyazaki, Ryotaro Kamohara, Go Hatachi, Hideyori Sengyoku, Hironosuke Watanabe, Tomohiro Obata, Laura E. Niklason, Takeshi Nagayasu

**Affiliations:** 1 Department of Surgical Oncology, Nagasaki University Graduate School of Biomedical Sciences, Nagasaki, 852-8501 Japan; 2 Translational Research Center, Research Institute for Science & Technology, Tokyo University of Science, Chiba, 278-8510 Japan; 3 Department of Radiation Medical Sciences, Atomic Bomb Disease Institute, Nagasaki University, Nagasaki, 852-8523 Japan; 4 Department of Cardiovascular Medicine, Translational Systems Biology and Medicine Initiative, Graduate School of Medicine, The University of Tokyo, Tokyo, 113-8654 Japan; 5 Center for Molecular Medicine, Jichi Medical University, Tochigi, 329-0498 Japan; 6 Department of Tumor and Diagnostic Pathology, Atomic Bomb Disease Institute, Nagasaki University, Nagasaki, 852-8523 Japan; 7 Department of Genome Repair, Atomic Bomb Disease Institute, Nagasaki University, Nagasaki, 852-8523 Japan; 8 Department of Genetics, Research Institute of Environmental Medicine, Nagoya University, Nagoya, 464-8601 Japan; 9 Department of Plastic Surgery, Wound Repair and Regeneration, Fukuoka University, Fukuoka, 814-0180 Japan; 10 FIRST Research Center for Innovative Nanobiodevices, Graduate School of Engineering, Nagoya University, Nagoya, 464-8603 Japan; 11 Medical-Engineering Hybrid Professional Development Center, Nagasaki University Graduate School of Biomedical Sciences, Nagasaki, 852-8501 Japan; 12 Department of Biomedical Engineering, Yale University, New Haven, CT 06520 USA; 13 Department of Anesthesia, Yale University, New Haven, CT 06520 USA

## Abstract

Bioengineered lungs consisting of a decellularized lung scaffold that is repopulated with a patient’s own cells could provide desperately needed donor organs in the future. This approach has been tested in rats, and has been partially explored in porcine and human lungs. However, existing bioengineered lungs are fragile, in part because of their immature vascular structure. Herein, we report the application of adipose-derived stem/stromal cells (ASCs) for engineering the pulmonary vasculature in a decellularized rat lung scaffold. We found that pre-seeded ASCs differentiated into pericytes and stabilized the endothelial cell (EC) monolayer in nascent pulmonary vessels, thereby contributing to EC survival in the regenerated lungs. The ASC-mediated stabilization of the ECs clearly reduced vascular permeability and suppressed alveolar hemorrhage in an orthotopic transplant model for up to 3 h after extubation. Fibroblast growth factor 9, a mesenchyme-targeting growth factor, enhanced ASC differentiation into pericytes but overstimulated their proliferation, causing a partial obstruction of the vasculature in the regenerated lung. ASCs may therefore provide a promising cell source for vascular regeneration in bioengineered lungs, though additional work is needed to optimize the growth factor or hormone milieu for organ culture.

## Introduction

Over 25 million adults in the United States alone are diagnosed with, or estimated to have, some form of chronic lung disease such as chronic obstructive pulmonary disease or idiopathic pulmonary fibrosis. Approximately 135,000 of these patients die from chronic lower respiratory disease annually^1, 2^. Lung transplantation is the only comprehensive treatment for prolonging survival and improving the quality of life for patients with end-stage lung disease^3^, but the number of donor lungs falls far short of the number needed, and lung transplantation is an expensive procedure with a low long-term survival rate^4^. For example, in the United States in 2014, approximately 2500 patients were placed on the lung transplantation waiting list, but suffered an overall mortality rate of 10.4%^5^. Furthermore, allograft rejection and infection remain unavoidable problems for transplant recipients, who must remain on immunosuppression for life^6^.

To address these complex transplantation problems, regenerative techniques are currently being developed to create autologous bioengineered versions of complex organs^7–11^. The procedure involves, generally, producing an acellular, organ-specific scaffold using detergents or enzymes to remove all cellular materials, and then recellularizing the organ using cells from the intended recipient. The major advantage of using a decellularized whole organ over other matrices is that the decellularized organ preserves the original three-dimensional (3D) architecture and complex natural extracellular matrix, which is critical for reconstructing hierarchical branching structures such as airways and vasculature. Using the recipient’s own cells for organ recellularization will also reduce or prevent immunological response to the implanted organ, at least theoretically.

Bioengineered organs based on such decellularized scaffolds have been explored in several laboratories^12–18^ using rodent and porcine models. However, in rat lung transplantation models, the function of the transplanted bioengineered organs regularly declines within several hours after transplantation. The transplanted lungs often fail owing to edema^13^, blood clotting, and alveolar hemorrhage^12^. These deficiencies are due, in part, to the incomplete repopulation of the lung microvasculature, with consequent permeability of the alveolar basement membrane, and exposed collagen which triggers coagulation.

In mammals, small blood vessels consist only of endothelial cells (ECs), whereas larger vessels are covered by mural cells: pericytes in smaller vessels, and smooth muscle cells (SMCs) in large vessels^19^. The association of mural cells with newly formed vessels is known to regulate EC proliferation, survival, migration, and differentiation, as well as barrier function and permeability. Therefore, in efforts to enhance lung function after transplantation, we investigated the impact of mural progenitor cells on the function of engineered microvessels within an engineered lung construct.

Mesenchymal stem/stromal cells (MSCs) are candidate therapeutic cells in regenerative medicine. Within this cell type, adipose tissue-derived stem/stromal cells (ASCs) are highly accessible and share many of the characteristics of their marrow counterparts, including extensive proliferative potential and the ability to undergo multi-lineage differentiation into mesenchymal cell types^20, 21^. In addition, ASCs may have a functional advantage, in terms of proliferative capacity and angiogenic potential, as compared to bone marrow-derived stromal cells (BMSCs)^22^. ASCs have been shown to have the ability to differentiate into a perivascular phenotype following EC contact or co-transplantation with ECs^23, 24^. In 3D fibrin matrices, ASCs stabilize EC networks by developing pericyte characteristics^25^, suggesting that adding ASCs to an engineered lung construct could promote the formation of mature vasculature in bioengineered lungs. Additionally, mesenchyme-targeting growth factors, especially fibroblast growth factor 9 (FGF9), is essential for the development of the embryonic lung^26^. FGF-9 stimulates the muscularization of neovessels and produces durable, vasoresponsive microvessels^27^.

The current study sought to test whether the stability of the pulmonary vasculature in bioengineered lungs could be improved by including ASC-derived mural cells within the engineered lung. We hypothesized that ASCs would differentiate into pericytes following interaction with the ECs, and that stimulation with FGF9 would tighten the EC-lined vasculature following pericyte coverage, thus regenerating a mature vasculature in the decellularized lung scaffold.

## Results

### Lung tissue engineering strategy

**Figure 1:**
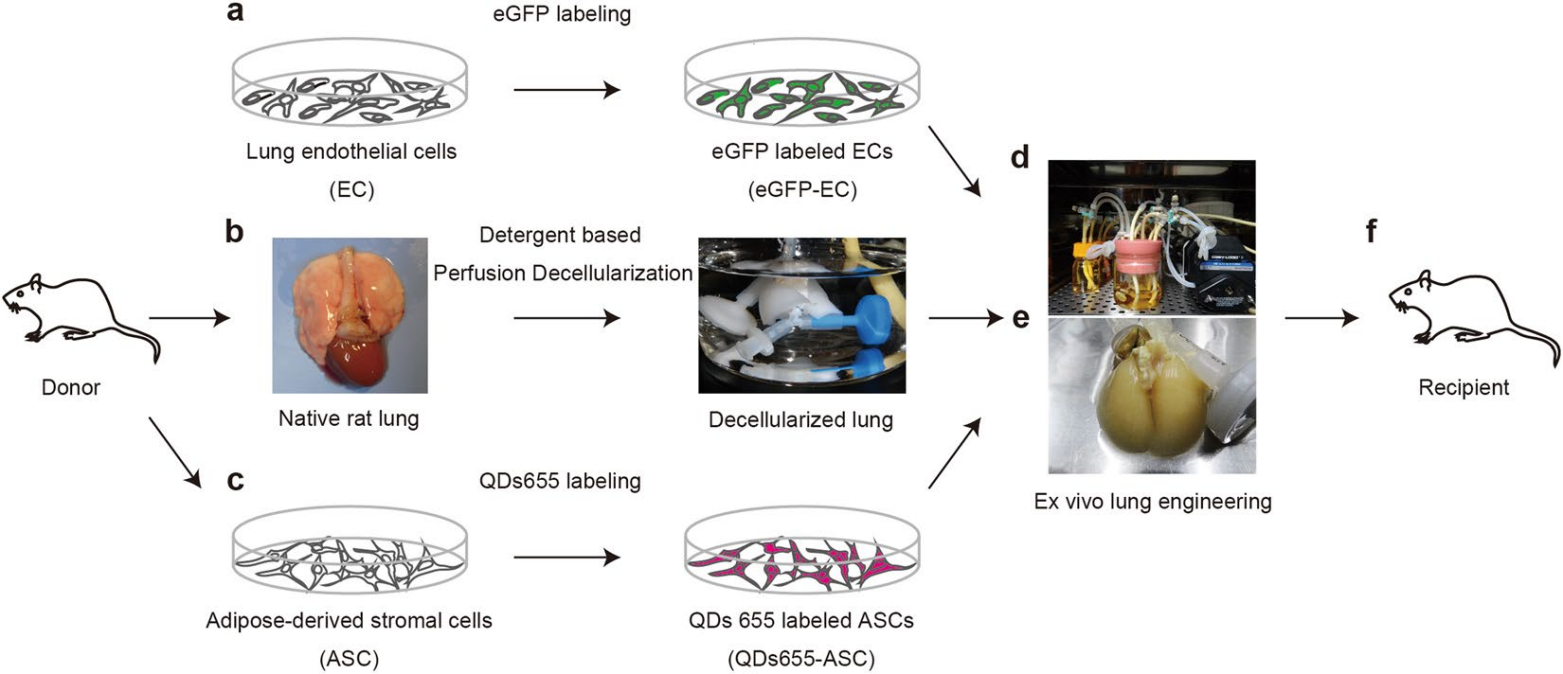
Schematic protocol for lung tissue engineering. (**a**) Donor rat lung microvascular endothelial cells (ECs) were cultured *in vitro* and labeled with enhanced green fluorescent protein (eGFP) by lentiviral infection. (**b**) Photographs of native and perfusion-decellularized rat lungs. Native rat lungs were cannulated in the pulmonary artery, vein, and trachea for infusion of decellularization solutions and decellularized by perfusion of SDS-based detergent. (**c**) Adipose-derived stromal cells (ASCs) were labeled with quantum dots 655 (QDs655) using octa-arginine peptide (R8), a cell-penetrating peptide. (**d**) The acellular matrix was seeded inside a biomimetic bioreactor with eGFP-labeled vascular ECs (eGFP-ECs) and QDs655-labeled ASCs (QDs655-ASCs). (**e**) Gross morphology images of white-colored decellularized lung and dark milky-white-colored recellularized lung after 8 days of culture. (**f**) After 8 or 16 days of culture, the regenerated lung was removed from the bioreactor for orthotopic transplantation into a syngeneic rat recipient, or for further analysis

To provide ECs for seeding the bioengineered lung, rat lung microvascular ECs were cultured and labeled with enhanced green fluorescent protein (eGFP) by lentiviral infection (Fig. 1a). The native rat lung was cannulated at the pulmonary artery (PA), pulmonary vein (PV), and trachea for infusion of sodium dodecyl sulfate (SDS)-based decellularization solutions (Fig. 1b). The ASCs that were isolated were labeled with quantum dots 655 (QDs655), a cell-penetrating peptide that consist of CdSe/ZnS-core/shell semiconductor nanocrystals (Fig. 1c). QD655 can be used for labeling ASCs while maintaining stem cell potency with low cytotoxicity. Labeling was performed here as previously described^28^.

Following lung organ decellularization, the acellular lung matrix was mounted inside a biomimetic bioreactor, thereby allowing the seeding of eGFP-labeled vascular ECs (eGFP-ECs) and QDs655-labeled ASCs (QDs655-ASCs) into the pulmonary vasculature, via anterograde PA perfusion (Fig. 1d). Three experimental groups, differing in cell composition and growth factors, were studied (n = 3 animals/group): (a) eGFP-ECs alone (EC group); (b) QDs655-ASCs with eGFP-ECs (EC–ASC group); or (c) a combination of QDs655-ASCs, eGFP-ECs, and FGF9 (EC–ASC–FGF9 group). These were done at 8 or 16 days after initial culture so that the effects of ASCs and FGF9 on EC survival *ex vivo* could be assessed (6 animals total/group). The gross appearance of the recellularized rat lung changed from white to a dark milky-white color after 8 days of culture (Fig. 1e) After 8 days of culture, the regenerated lung was removed from the bioreactor for orthotopic transplantation into the syngeneic rat recipient (Fig. 1f), or for further analysis.

### Pulmonary vascular architecture was preserved in the decellularized rat lung scaffolds

**Figure 2:**
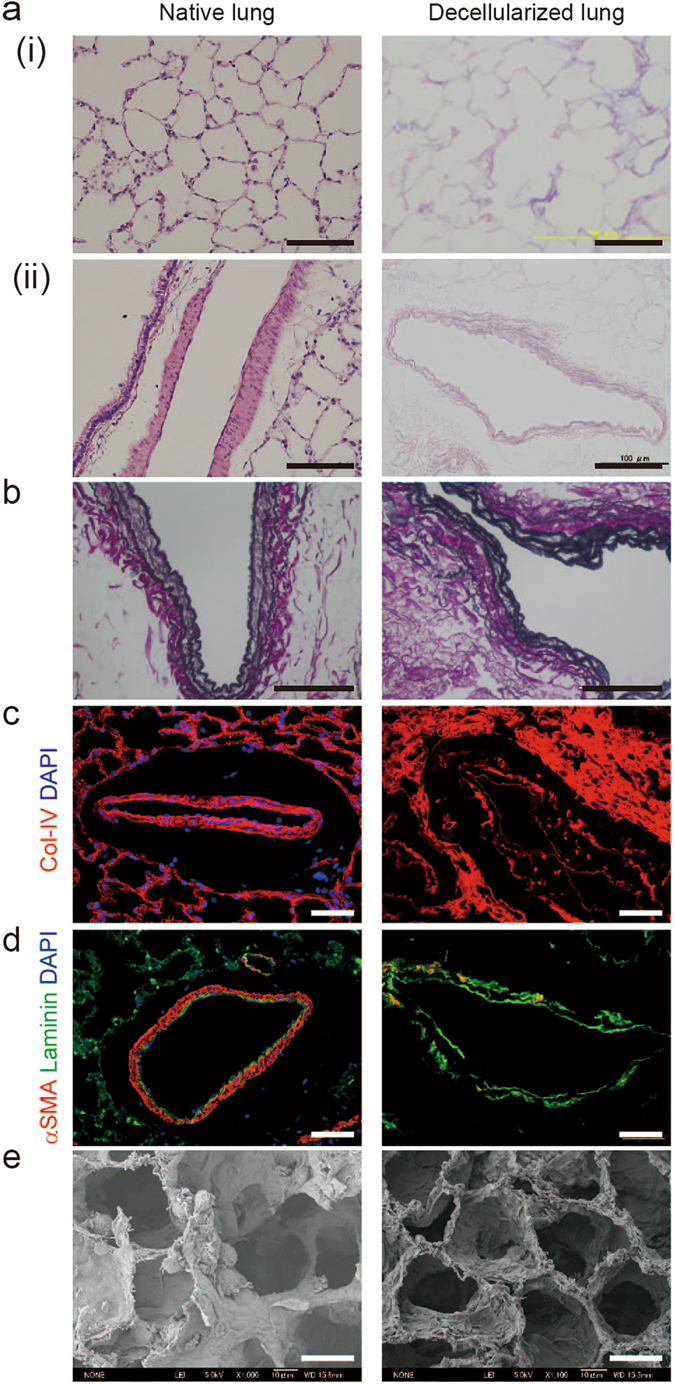
Decellularization of whole rat lungs generates a pulmonary vasculature matrix without a cellular component. Thin sections from native (left panels) and decellularized (right panels) rat lungs: (**a**) hematoxylin and eosin **(**H&E) staining showing no remaining nuclei or intracellular components in the decellularized lung parenchyma (i) or pulmonary vasculature (ii); (**b**) Elastica van Gieson staining showing pulmonary vasculature elastic fibers in the decellularized lungs; (**c, d**) Fluorescence micrographs showing preservation of the pulmonary vasculature matrix after decellularization comprising (**c**) collagen IV (red) and (**d**) laminin (green), with no remaining cellular components (**c, d**: DAPI, blue) or α-smooth muscle actin (**d**: red); (**e**) scanning electron microscopy images showing preservation of the 3D decellularized matrix scaffold structure. Scale bars: 100 μm (**a–d**); 20 μm (**e**).

To confirm that the lung scaffolds were adequately decellularized while preserving matrix structure, we characterized decellularized scaffolds using histological and immunofluorescence staining of collagen-IV, laminin, and ɑ-smooth muscle actin (ɑ-SMA) (Fig. 2a–d). Elastica van Gieson staining revealed the preservation of elastic fibers (Fig. 2b). The immunofluorescence staining revealed that collagen-IV and laminin were preserved, but ɑ-smooth muscle actin was not preserved. Scanning electron microscopy^29^ confirmed that alveolar ducts and an alveolar network were completely preserved in the absence of cellular components in the decellularized lung (Fig. 2e). These results suggest that the rat lungs were fully decellularized and retained the structural elements needed to support the growth of a reseeded lung.

### Primary ASCs (P0) comprise MSC-lineage cells, including pericytes and SMCs

**Figure 3:**
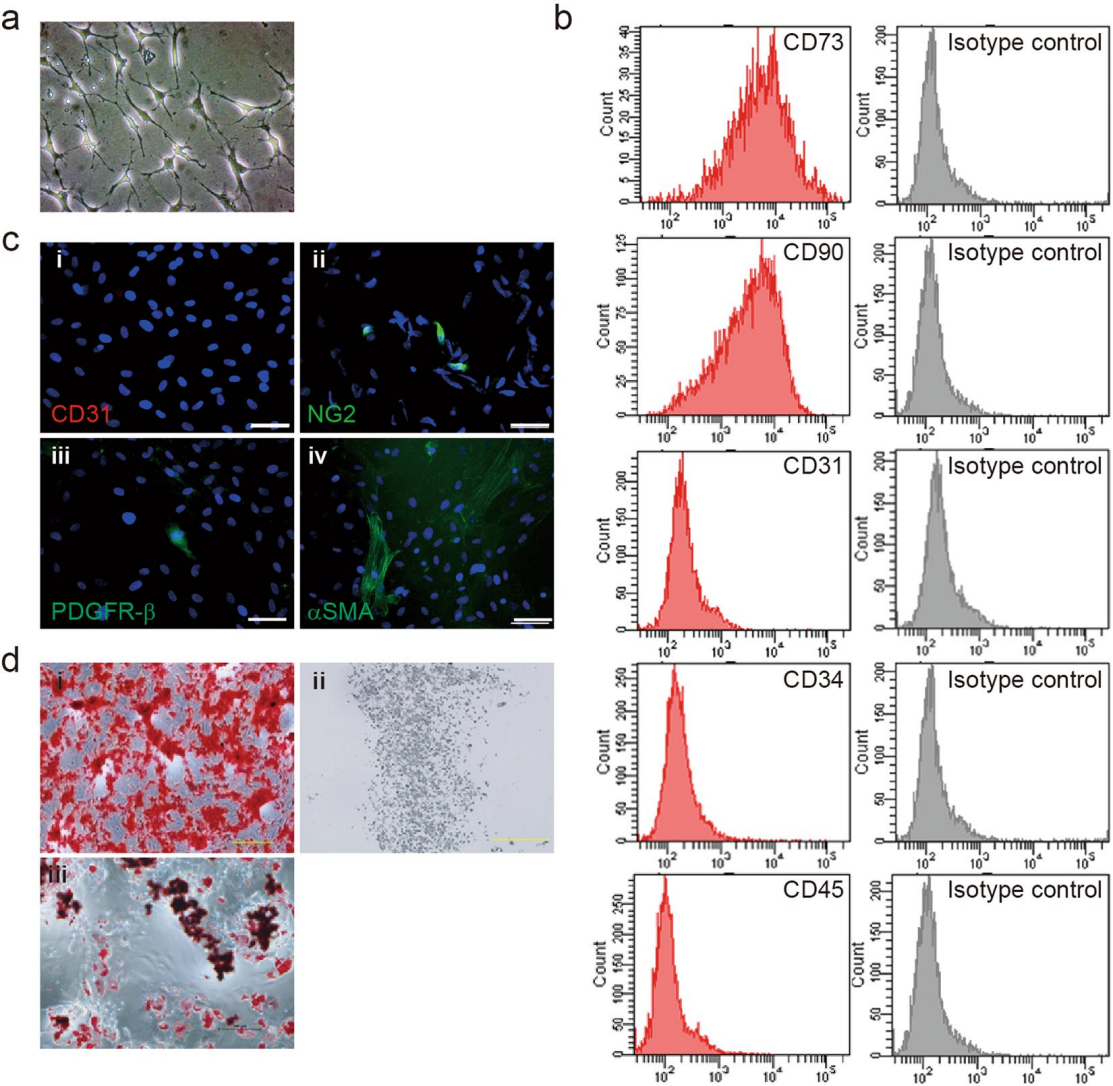
ASCs comprise mostly mesenchymal stem/stromal cells (MSCs) with pericytes and smooth muscle cells. (**a**) Phase-contrast image of ASCs 3 days after initial plating (passage 0). (**b**) FACS analysis of ASCs measuring the expression of MSC markers 5 days after initial plating. Passage-0 ASCs expressed the MSC markers CD73 and CD90 and lacked endothelial CD31 and hematopoietic CD34 and CD45 cell markers. (**c**) Immunofluorescence analysis showed that ASCs lacked the EC marker CD31 (i, red) and expressed the pericyte markers chondroitin sulfate proteoglycan 4 (NG2; ii, green), platelet-derived growth factor receptor beta (PDGFR-β; iii, green) and smooth muscle actin (SMA; iv, green). Scale bars: 50 μm (i–iv). (**d**) Multi-lineage potential of passage-0 ASCs *in vitro*. ASCs were analyzed for osteogenic (i, Alizarin Red S), chondrogenic (ii, Alcian Blue), and adipogenic (iii, Oil Red O) differentiation capacity.

We next isolated rat syngenic ASCs from subcutaneous fat tissue as previously described^20^. At day 5 of ASC culture, most of the hematopoietic cells were lost, and the percentage of plastic-adherent ASCs increased (Fig. 3a), consistent with previous studies^30^. Flow cytometric analysis of cell surface marker expression revealed that the plastic-adherent cells exhibited a similar phenotypic profile to stromal cells (CD73+, CD90+) but not hematopoietic cells (CD31−, CD45−) (Fig. 3b). The expression of the pericyte/SMC markers chondroitin sulfate proteoglycan 4 (NG2), platelet-derived growth factor receptor beta (PDGFR-β), and α-SMA were scant by immunofluorescence staining (Fig. 3c), suggesting most of the cultured ASCs were not differentiated into a pericyte/SMC lineage. The cultured ASC populations expressed characteristic multi-potential properties, as shown by their capacity to form Alizarin Red-positive mineralized deposits, Oil Red O-positive lipid droplets, and Alcian Blue-positive proteoglycan-rich matrix *in vitro* after suitable induction with known adipogenic and chondrogenic factors (Fig. 3d). Taken together, these results suggest that the quality of the isolated ASCs warranted further examination within the context of the engineered lung vasculature.

### Seeded ASCs surrounded the endothelium in the perivascular space

**Figure 4:**
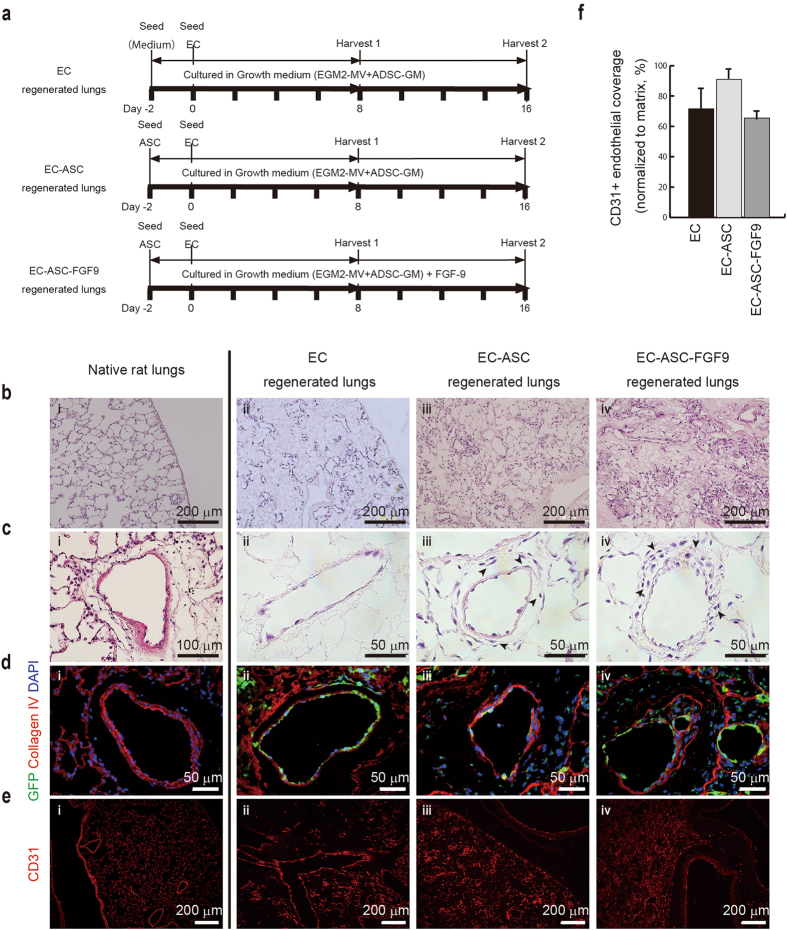
ASCs enhanced endothelium coverage, localized beside the endothelium monolayer, and migrated into the vascular adventitia and stroma in acellular scaffolds at day 8 of the culture. (**a**) Schematic recellularization protocol. EC-regenerated lung repopulated with eGFP-ECs alone; EC–ASC-regenerated lung repopulated with a combination of eGFP-ECs and QDs655-ASCs; EC–ASC–fibroblast growth factor 9 (FGF9)-regenerated lung repopulated with a combination of eGFP-ECs and QDs655-ASCs and cultured in EGM2-MV/ADSC-GM with FGF9. (**b,c**) H&E stained low- (**b**, x10) and high- (**e**, x40) magnification views of thin sections from native (i), EC- (ii), EC–ASC- (iii), and EC–ASC–FGF9- (iv) regenerated lungs. ECs distributed throughout the lung parenchyma (**c**, ii) and formed a complete endothelium monolayer in the EC-, EC–ASC-, and EC–ASC–FGF9-regenerated lungs. Other cells (black arrowheads, ECs or ASCs) localized to the tunica adventitia of the pulmonary vasculature in the EC–ASC- and EC–ASC–FGF9-regenerated lungs. (**d**) Immunofluorescence micrographs of thin sections from native (i) and regenerated (ii–iv) lungs showing the expression of collagen-IV and engraftment of GFP-ECs. ASCs (stained with DAPI alone) engrafted both in the basement membrane and in the tunica adventitia in the EC–ASC- and EC–ASC–FGF9-regenerated lungs. (**e**) Immunofluorescence micrographs of thin sections from native (i) and regenerated (ii–iv) lungs showing expression of CD31. (**f**) Quantification of endothelial coverage in EC-, EC–ASC-, and EC–ASC–FGF9-regenerated lungs after 8 days in culture. All values are means ± standard deviation^54^ of three independent experiments. *P* = 0.0554 (ANOVA); *P* = 0.1767 (Kruskal–Wallis test).

ASCs secrete a variety of angiogenic cytokines and play a supportive role in vascular regeneration by differentiating into pericytes or smooth muscle cells^31–33^, with FGF9 facilitating vascular maturation^27^. To determine the supportive functions of ASCs on ECs in the decellularized lung scaffold, we evaluated the three recellularization models described above: ECs alone (EC group); ASCs and ECs (EC–ASC group); and ASCs, ECs and FGF9 (EC–ASC–FGF9 group). We maintained the cell-seeded lungs for either 8 or 16 days in isolated organ culture (Fig. 4a).

Eight days after recellularization, hematoxylin and eosin (H&E) staining of recellularized lungs showed a greater number of cells in the EC–ASC and EC–ASC–FGF9 groups than in the EC group (Fig. 4b). Detailed histological examination revealed ECs inside the pulmonary vasculature and capillary matrix in all three groups (Fig. 4c, ii–iv, Supplementary Fig. 1). Fibroblast-like, spindle-shaped cells were observed in the perivascular space in the EC–ASC and EC–ASC–FGF9 groups, suggesting that ASCs also infiltrated the stroma (Fig. 4c, iii, iv).

To track the fact of ECs within the matrix, the lung scaffolds were re-endothelialized with eGFP-labeled ECs (eGFP-ECs). Dispersion and engraftment of eGFP-ECs were observed throughout the pulmonary vascular scaffold (Fig. 4d). Immunofluorescence imaging of eGFP-ECs and collagen IV in 8-day-regenerated lungs revealed eGFP-ECs embedded on alveolar basement membrane (Fig. 4d), with ASCs (as visualized by 4′,6-diamidino-2-phenylindole [DAPI] staining alone) localized in the perivascular regions (Fig. 4d, iii, iv). We therefore surmised that the perivascular spindle-shaped cells, which did not stain for eGFP, were ASCs. Additionally, ASC engraftment was increased when FGF9 was administrated with ECs and ASCs during recellularization (Fig. 4d, iv). Immunofluorescence staining of the endothelial marker CD31 showed that the seeded ECs were distributed throughout the large vessels and through to the capillaries (Supplementary Fig. 1) and appeared to form a monolayer. EC coverage within the scaffold did not differ between the groups at day 8 of culture (Fig. 4e,f).

### ASCs differentiated into pericytes and wrapped around pulmonary vessels in the regenerated lung

**Figure 5:**
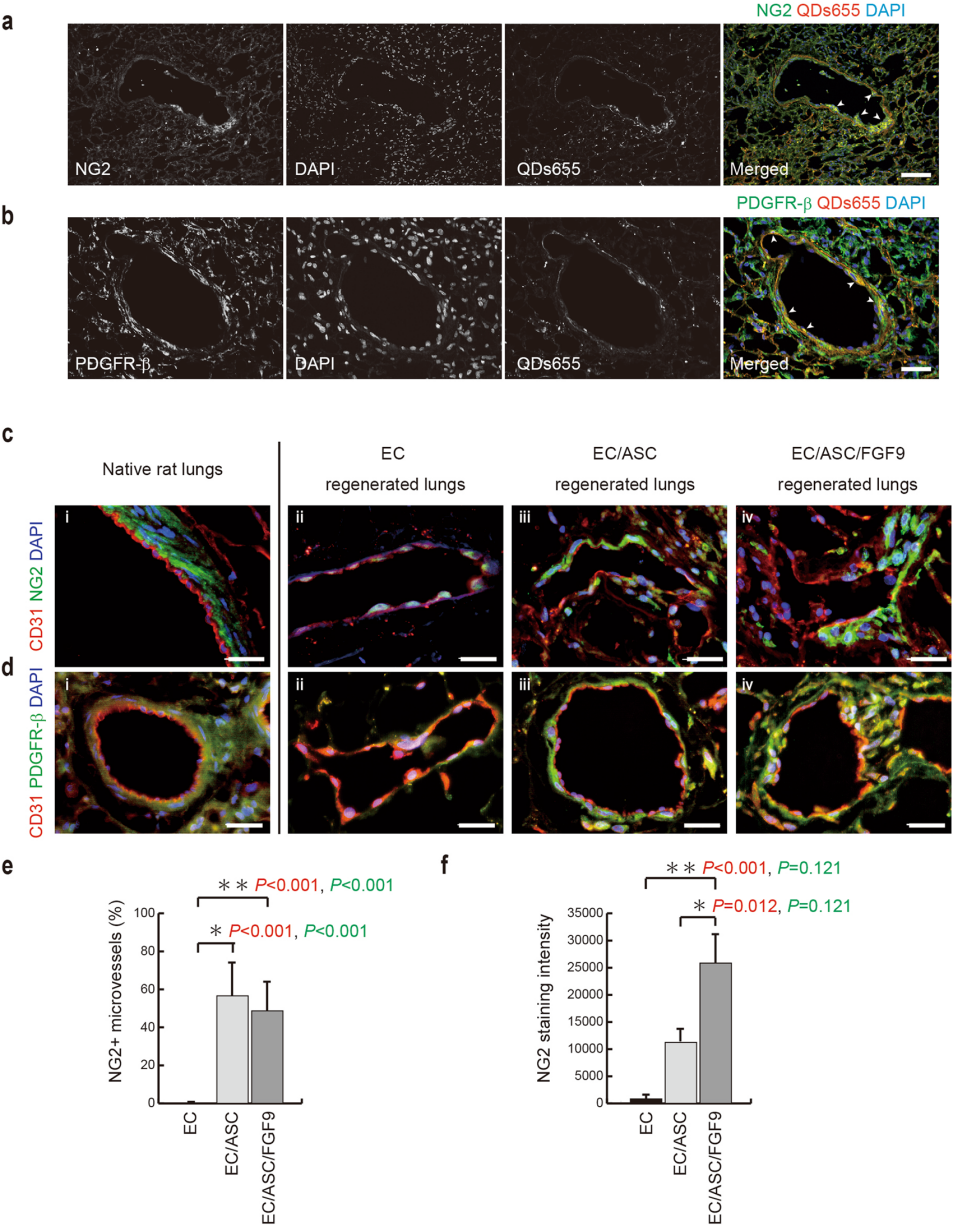
ASC differentiation into pericytes, supported by FGF9, stabilized pulmonary vessels in the regenerated lung. (**a,b**) Corresponding immunofluorescence micrographs of thin sections from EC–ASC-regenerated lungs (day 8 of culture) showing expression of NG2 (**a**, green) and PDGFR-β (**b**, green) in quantum dots 655 (QDs655)-labeled (red) ASCs. Nuclear DAPI (blue). QDs655-labeled ASCs were clearly localized to the vascular wall and expressed pericytic markers. (**c,d**) Immunofluorescence micrographs of thin sections from native (i) and regenerated lungs (ii–iv, day 8 of culture) showing expression of CD31 (red) with PDGFR-β (**c**, green) or CD31 (red) with NG2 (**d**, green) around the pulmonary vasculature. Note the perivascular areas of NG2- or PDGFR-β-positive cells are thicker in the EC–ASC–FGF9 group. Nuclear DAPI (blue). Scale bars: 100 μm (**a**) and 50 μm (**b–d**). (**e**) Bar graph showing the rate of NG2^+^ pulmonary vessel formation relative to total pulmonary vessels within the regenerated lung parenchyma. Comparison of all groups; *P* < 0.001 (ANOVA); *P* < 0.001 (Kruskal-Wallis test). (**f**) Bar graph showing NG2 staining intensity within the regenerated lung parenchyma. Comparison of all groups; *P* *<* 0.001 (ANOVA); *P* = 0.027 (Kruskal-Wallis test). Comparison of two groups in panels (**e**) and (**f**); the p-values of Bonferroni test and Steel-Dwass test are shown in red and green, respectively. All values are means ± SD of three independent experiments.

To assess whether ASCs differentiated into pericytes in our organ culture system, we performed immunofluorescence staining for NG2 and PDGF-β in the regenerated lungs at day 8 of culture. Cell tracking using quantum dots in the EC-ASC group (QDs655) revealed that NG2 or PDGFR-β-positive cells were present among the QDs655-labeled ASCs (Fig. 5a,b). Immunofluorescence staining revealed NG2- or PDGFR-β-expressing microvessels in the EC–ASC and EC–ASC–FGF9 groups. Expression of NG2 and PDGFR-β was not observed in the EC group at day 8 of culture (Fig. 5c,d). Furthermore, the thickness of the perivascular NG2- or PDGFR-β-positive cell layer was thicker in the EC–ASC–FGF9 group as compared to the EC-ASC group. The percentage of NG2-positive cell-wrapped microvessels was similar in the EC–ASC and EC–ASC–FGF9 groups (Fig. 5e). However, the NG2 staining intensity in the EC–ASC–FGF9 group appeared to be double that of the EC–ASC group at day 8 of culture (Fig. 5f). Taken together, these results suggest that ASCs differentiate into pericytes in this system, and that FGF9 up-regulated cell proliferation and/or this differentiation fate.

### ASCs facilitated *ex vivo* EC survival in regenerated lungs

**Figure 6:**
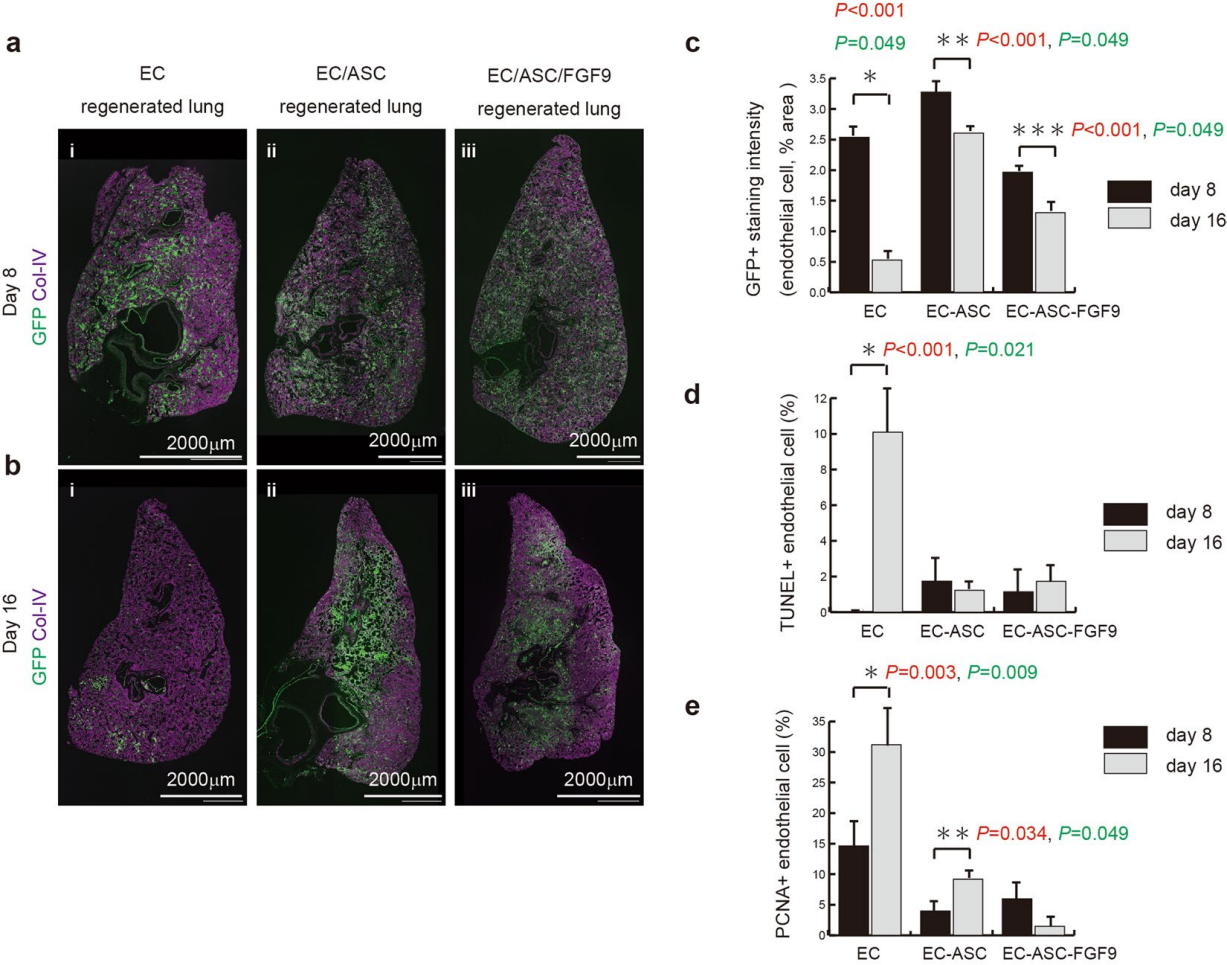
ASCs facilitate *ex vivo* EC survival in regenerated lungs. (**a, b**) Grouped immunofluorescence images of whole EC- (i), EC–ASC- (ii) and EC–ASC–FGF9- (iii) regenerated lungs showing expression of eGFP-ECs (green) and collagen IV (purple) at days 8 (**a**) and 16 (**b**) of culture. Gross distribution of eGFP-ECs was similar in each group at day 8 (**a**), and the majority of eGFP-ECs had disappeared by day 16 in the EC group (**b, i**). Scale bars: 2000 μm. (**c**) Bar graph showing expression of eGFP staining intensity normalized to collagen IV matrix at days 8 and 16 of culture. (**d**) Bar graph showing the rate of TUNEL^+^ ECs relative to total ECs within a unit area of the regenerated lungs (at days 8 and 16 of culture). (**e**) Bar graph showing the rate of PCNA^+^ ECs relative to total ECs within a unit area of the regenerated lungs (at days 8 and 16 of culture). The p-values by the Student’s t-test and Mann–Whitney U test are shown in red and green, respectively. All values are means ± SD of three independent experiments.

We next investigated whether ASCs could prolong the survival of ECs within the engineered pulmonary vessels. Immunofluorescence imaging of eGFP-ECs and collagen IV in the regenerated lungs revealed that the gross distribution of eGFP-ECs was similar among the groups at day 8 (Fig. 6a). However, a majority of eGFP-ECs were lost in the EC group at day 16 (Fig. 6b). Indeed, eGFP-EC staining intensity, normalized to collagen IV per unit area, was significantly diminished in the EC group at day 16 (Fig. 6c). Complementary analysis using terminal deoxynucleotidyl transferase dUTP nick end labeling (TUNEL) and proliferating cell nuclear antigen (PCNA) assays was used to assess the relative rates of apoptosis and cell division, respectively. In the EC group, TUNEL assay showed that 10.1% of the ECs were undergoing apoptosis at day 16 in culture. However, EC apoptosis was strongly suppressed in the other groups (Fig. 6d, Supplementary Fig. 2). Interestingly, the PCNA assay indicated a greater number of dividing cells in the EC group than in the other groups (Fig. 6e, Supplementary Fig. 3), suggesting an increased turnover of ECs. Taken together, this analysis showed that the number of dying cells exceeded the number of proliferating cells in the EC group, and that the addition of ASCs apparently prevented this by promoting a quiescent state in the ECs, and prolonging their survival in the engineered pulmonary vessels.

### ASC gene expression profile in the regenerated lungs

To assess whether ASCs provide angiogenic factors in the context of these bioengineered lungs, we investigated the expression profile of angiogenesis-related genes in EC–ASC- and EC-regenerated lungs at day 8 of culture using reverse transcription quantitative real-time polymerase chain reaction (qRT-PCR) (Supplementary Table 1). The mRNA levels of angiopoietin-1 (ANG1), a pericyte-secreted cytokine that regulates angiogenesis and vascular sequencing, increased significantly (18.85-fold) in the presence of ASCs. mRNA levels of ASC-secreted angiogenic cytokines, including hepatocyte growth factor (HGF), vascular endothelial growth factor-alpha (VEGF-A), and c-fos induced growth factor (FIGF), were also significantly increased. In contrast, the mRNA levels of TEK tyrosine kinase (Tie-2/Tek) and TIE domains 1 (Tie-1), which play critical roles in angiogenesis, significantly decreased in the presence of ASCs. Interestingly, the mRNA levels of transforming growth factor-beta 2 (TGF-β2) and its receptor, endoglin, both decreased. Furthermore, matrix metalloproteases 2 (MMP2) and 14 (MMP14), which degrade the major structural components of the basement membrane, including type IV collagen, were highly upregulated (7016.41- and 17.27-fold, respectively) in the presence of ASCs. Finally, alanyl aminopeptidase (Anpep), which promotes endothelial invasion, was also highly upregulated (839.38-fold) in the presence of ASCs. Overall, these results indicate that the gene expression profiles reflected the histological findings.

### ASC–EC interactions induced vasculature network maturation with physiological PA pressure

**Figure 7:**
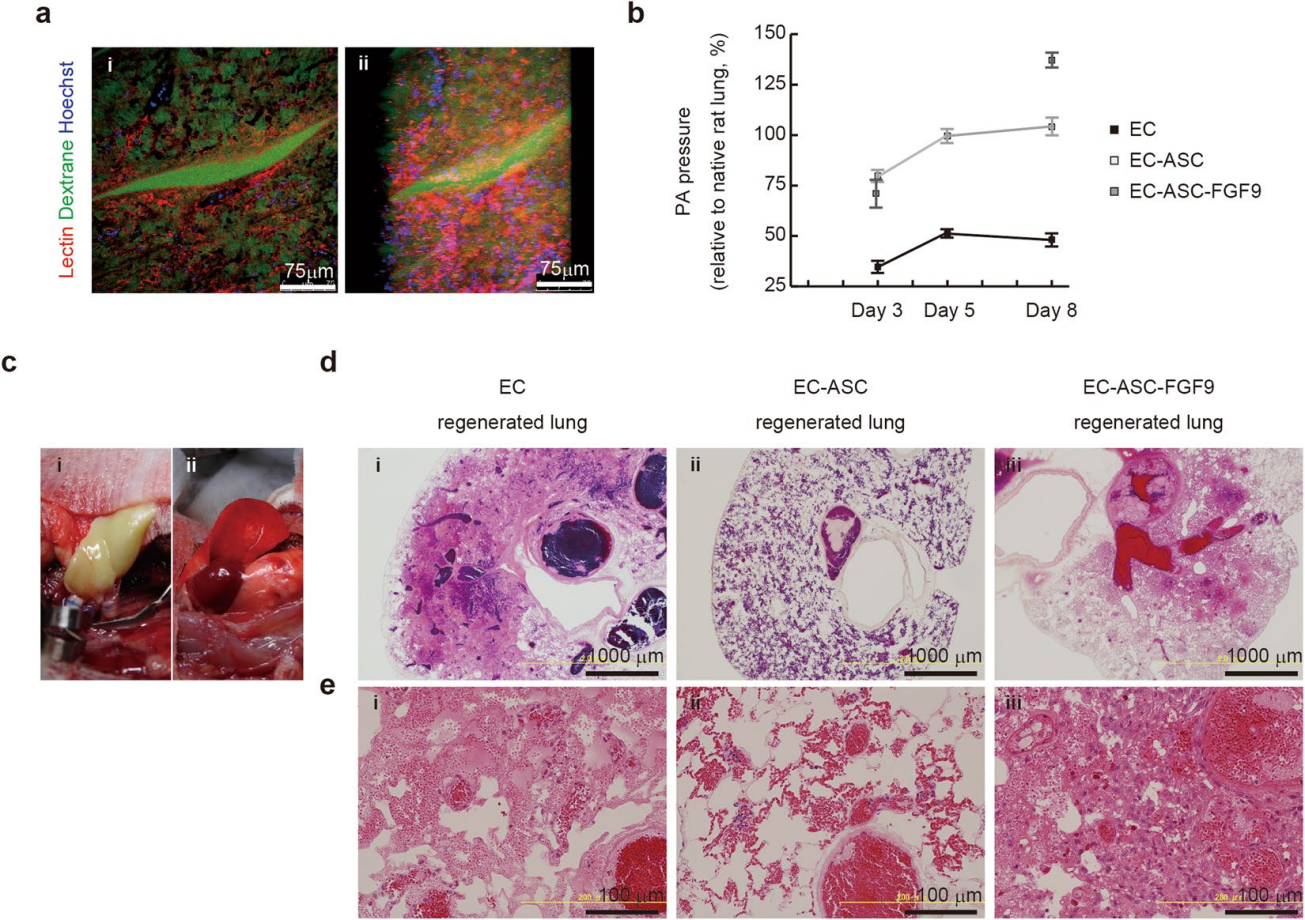
ASC–EC interaction induces development of a vascular network with physiological PA pressure, suppressing lung edema and alveolar hemorrhage of regenerated lungs *in vivo*. (**a**) Multi-photon excitation microscopy images (i) and 3D reconstruction (ii) of EC–ASC regenerated lungs (day 8 of culture) showing the lectin-labeled pulmonary capillary network (red) in the FITC-dextran-labeled lung matrix (green). When seeded with ASCs, ECs assembled into a capillary network, representing native lung parenchyma. (**b**) Line chart summarizing sequential PA pressures of regenerated lungs under constant rate perfusion (4 mL/min) at days 3, 5, and 8 (normalized to native rat lung pressure). (**c**) Orthotopic transplantation of EC–ASC-regenerated lungs. Representative pictures showing a regenerated lung graft after anastomosis of the pulmonary artery, pulmonary vein, and bronchus before (i) and after re-perfusion (ii). All engineered lungs were perfused with blood over a period of seconds to minutes. (**d, e**) Corresponding H&E-stained low- (**d**, x4) and high-magnification (**e**, x20) views of thin sections from regenerated lungs (i–iii) 3 h after transplantation. In the EC group, exudative fluid leaked into the alveolar space, representing lung edema. In the EC–ASC group, fluid leakage was suppressed and red blood cells were retained in the lung vessels and alveolar capillaries. In the EC–ASC–FGF9 group, regenerated cells grew into the vascular space obstructing the blood supply. All values are means ± SD of three independent experiments.

To assess whether the ASC–EC-regenerated lungs could develop a capillary network, we performed fluorescence microangiography of the regenerated lungs using multiphoton laser scanning microscopy. When the ECs and ASCs were seeded together, the ECs assembled into a capillary network representative of the native lung parenchyma (Fig. 7a). To evaluate the patency of the vascular channels, we measured PA pressure at a flow rate of 4 mL/min in the pulmonary artery (Fig. 7b). Interestingly, the average PA pressure of the EC group (8.60 ± 3.43 mmHg) was less than half that of the native lung (17.66 ± 1.60 mmHg), suggesting hydrostatic pressure loss due to transport across the alveolar membrane, as reported previously^34^. Strikingly, the EC–ASC group showed a similar PA pressure (18.69 ± 2.64 mmHg) to that of the native lung at day 8. The EC–ASC–FGF9 group showed a time-dependent increase in PA pressure, which exceeded 125% of that of the native lung at day 8 (23.75 ± 2.59 mmHg). The result indicated the occlusive change of vascular channels in EC–ASC–FGF9 group.

### ASC–EC interactions suppress lung edema and alveolar hemorrhage in regenerated lungs

To assess the integrity of the bioengineered pulmonary vasculature *in vivo*, we performed orthotopic transplantation of left lungs that had been recellularized for 8 days. (Fig. 7c). Using the cuff technique^35^, all vessels and bronchi were connected between the engineered and recipient airways and vasculature. After transplantation, engineered lungs were ventilated with no visible air leaks from the parenchyma. All engineered lungs perfused with blood over a period of seconds to minutes after release of the vascular clamps (Fig. 7c, ii). After transplantation, the recipient rats were extubated and observed for up to 3 h, after which time the animals were euthanized and the implanted lungs harvested.

Consistent with previous reports^12, 13^, clots were noted among all regenerated lungs at 3 h after transplantation (Fig. 7d). However, the pathological features of the transplanted lungs differed between groups. In the EC group, exudative fluid and red blood cells leaked into the alveolar space, representing lung edema and alveolar hemorrhage, respectively (Fig. 7d,i,7e,i). In the EC–ASC group, the fluid leakage was suppressed, and red blood cells were retained in the lung large vessels and alveolar capillaries (Fig. 7d,ii,e,ii). In the EC–ASC–FGF9 group, high cell proliferation obstructed the blood flow and likely contributed to alveolar hemorrhage, especially in the central area of the regenerated lungs (Fig. 7d,iii,e,iii). We therefore concluded that, overall, EC–ASC co-seeding resulted in somewhat more functional engineered vasculature as compared to EC or to EC-ASC-FGF9 conditions.

## Discussion

This study is the first to demonstrate revascularization of a decellularized lung scaffold using rat ASCs and ECs. Most attempts to induce revascularization of decellularized lung scaffolds have been based on endothelialization only, without the introduction of supportive mural cells. However, *in vivo*, vascular development and angiogenesis are achieved by a series of steps, including endothelial lumen formation and recruitment of perivascular cells. Mesenchymal stem cells (MSC) play a variety of roles during tissue vascularization, through both direct cell-cell contact and through indirect signaling, including the secretion of cytokines, growth factors, and extracellular matrix proteins^36^. Co-culture of ASCs with ECs promotes ASC differentiation towards the vascular SMC lineage^24, 37^. Consequently, the primary aim of the present study was to determine whether ASC co-cultivation with ECs could facilitate the perivascular coverage of developing nascent vessels and enhance EC survival in the decellularized rat lung scaffolds, thus improving the formation of a bioengineered pulmonary vasculature.

We present several lines of evidence showing that ASC co-cultivation with ECs enhances the formation of the pulmonary vasculature. First, we demonstrated that ASCs migrate to and accumulate around the nascent endothelial lumens, and differentiate from a progenitor phenotype toward a pericyte lineage. Furthermore, some ASCs migrated into the tunica adventitia of larger vessels in the lung scaffold, and may have behaved similar to vessel-resident stem and progenitor cells of native pulmonary vasculature. Second, time-series analysis of the regenerated lung showed that ASCs supported EC survival and reduced cell turnover at the 16 day time point, and promoted a quiescent EC state. Similar to the organization of native vasculature^38^, the ASC-derived pericytes stabilized the nascent engineered pulmonary. Thus, our results indicate that ASCs may provide a perivascular cell source that can stabilize vascular assembly in decellularized lung scaffolds.

Regarding aspects of feeding and angiogenesis of ASC, we determined the angiogenesis-related gene expression profiles in EC and EC-ASC regenerated lungs. The qRT-PCR data suggested that ASCs stimulated angiogenesis, maintained vascular maturation, and enhanced matrix remodeling in the decellularized scaffold. Gene expression profiling revealed significantly higher gene expression of components of the angiogenesis-related ANG/TIE signaling system and integrin/protease markers in the EC–ASC group as compared to the EC group. Analysis of the secretion profile revealed that key angiogenesis-related mediators, such as VEGF-A, HGF, and FIGF were upregulated, consistent with previous reports^39–42^. Furthermore, ANG-1 gene expression was strongly upregulated. ANG-1 is expressed by mural cells and stimulates mural coverage and basement membrane deposition, thereby promoting vessel tightness^13^. In contrast, Tie1 and Tie2/Tek, which are essential for EC proliferation, migration, and survival during angiogenesis, were down-regulated in EC-ASC cultures. This implies improved quiescence of the co-culture system as compared to the EC-only cultures. Thus, ASCs potentially promote the maturation of vessels rather than the induction of angiogenesis. However, confounding this interpretation is the fact that Anpep and proteases were also upregulated in the presence of ASCs^37, 43^.

We also investigated whether FGF9 could induce mural cell coverage in regenerating vessels. Immunohistochemical analysis of the regenerated lungs revealed an increase in recruited pericytes and a higher proportion of microvessels with pericytes in the EC–ASC–FGF9 group as compared to the EC-ASC group, consistent with a previous report^27^. However, FGF9 at this concentration seemed to overstimulate ASC growth, thereby inducing vascular obstruction. In the present study, a single dose of FGF9 was administered (50 ng/mL). Timed treatment or modulated release studies might be necessary to determine the optimum conditions for the beneficial effects of FGF9 in this system.

The transplanted bioengineered lungs displayed several intriguing pathological features. In the EC group, the regenerated lung exhibited typical pulmonary edema and alveolar hemorrhage 3 h after transplantation, indicating that vascular coverage was incomplete and that a vascular barrier had not been established. In the EC-ASC group, alveolar hemorrhage was not observed, indicating that vascular coverage was achieved and that a vascular barrier to cellular blood elements was present. Surprisingly, in the EC-ASC-FGF9 group, excessive ASC proliferation resulted in vessel obstruction, with consequent probably rises in pulmonary arterial pressures and alveolar hemorrhage. Accordingly, *ex vivo* pulmonary artery (PA) pressures were low in the EC group, and comparatively high in the EC-ASC-FGF9 group, as compared with native lung.

In a recent study, Ren and colleagues used induced pluripotent stem cell-derived ECs and pericytes for lung re-endothelialization using a two-phase culture protocol^34^. Passage-0 ASCs were used in the current study as a mural cell source because ASCs contain a unique population of immature cells with proangiogenic potential^44^. We propose native/autologous ASCs as an ideal pericyte cell source for vasculature regeneration, because they can be readily harvested and genetic modification is not necessary before their utilization. However, given that ASCs enhanced extracellular matrix remodeling in long-term culture^45^, which might lead to undesirable breakdown of the alveolar basement membrane, optimized strategies to control protease activity are essential for future applications. Application of a two-phase culture protocol, including a phase 1 angiogenic medium and a phase 2 stabilization medium, may be effective at suppressing nonessential matrix remodeling during lung regeneration^34^.

The lack of gas exchange analysis to confirm lung functionality *ex vivo* and/or *in vivo* is a limitation of this study. The majority of published work on lung tissue engineering, especially on the recellularization of decellularized lung scaffolds, has focused on the airway compartments. However, studies attempting synchronized re-epithelialization and re-endothelialization using alveolar cells and vascular cells have not been fully successful, possibly because of the complexity of the experimental design. Therefore, we propose that epithelial recellularization should be performed after the achievement of mature vascular regeneration. Furthermore, complete recellularization may require long-term organ culture with growth factors or hormones^46^.

In conclusion, we have shown that ASCs differentiate into perivascular cells that can stabilize the nascent pulmonary vasculature and migrate into the vascular adventitia in acellular rat lung scaffolds. Gene expression profiling of the regenerated lungs revealed that the ASCs provided several angiogenic factors, which resulted in EC survival and vessel maturation, thereby decreasing alveolar hemorrhage post-transplantation. The present study supports ASCs as an attractive cell source for pulmonary vasculature engineering.

## Methods

### Study approval

All animal experiments were approved by the Nagasaki University Institutional Animal Care and Use Committee (study number T-1010) and performed in compliance with the Animal Welfare Act.

### Perfusion decellularization of rat lungs

Rat donor lungs were obtained from young adult male Fischer 344 rats (8–12 weeks old, CLEA Japan, Tokyo). Lung decellularization was performed in accordance with previous reports^12, 13, 47, 48^. Briefly, animals were anesthetized with an intraperitoneal injection of ketamine/xylazine. After systemic heparinization, a median sternotomy was made with a transverse abdominal incision, just below the costal margin, allowing opening of the pericardium and bilateral pleural spaces. Immediately after ventriculotomy, the lungs were perfused via the right ventricle with phosphate-buffered saline (PBS) containing heparin (50 units (U)/mL) and sodium nitroprusside (1 µg/mL) (Sigma-Aldrich, MO) and were cleared of blood *in situ*. The lungs, heart, and trachea were then removed *en bloc*. The PA and trachea were directly cannulated, the PV was cannulated through the left atrial appendage, and the aorta was ligated. The freshly harvested rat lungs were connected to a perfusion system through the PA and trachea, and were mounted in a lung decellularization bioreactor. The lungs were gravity-perfused via the PA at physiologically appropriate PA pressures (below 20 mmHg). The lungs were perfused sequentially with heparinized (50 U/mL) PBS (100 mL) containing calcium/magnesium for 5 min, 0.0035% Triton-X-100 (Sigma) in deionized water for 10 min, and 250 ml of deoxyribonuclease reaction buffer containing magnesium chloride. The lungs were inflated with benzonase endonuclease (90 U/mL) and incubated for 1 h at room temperature before SDS perfusion. The lungs were then decellularized by sequential perfusion with 1 M NaCl and a concentration gradient (0.01% to 0.1%) of SDS in deionized water, followed by 1% Triton-X-100 in deionized water. After decellularization, tissues were rinsed extensively with PBS to remove residual detergent and cellular debris, perfused with PBS containing penicillin (100 U/mL), streptomycin (100 U/mL; both Invitrogen) and amphotericin B (2.5 mg/L; Sigma) and stored at 4 °C until further use.

### Bioreactor design

The lung regeneration bioreactor was based on previous publications^12, 47^. All bioreactor components were obtained from Cole-Parmer (Vernon Hills, IL). A silicone stopper and 500-mL glass jar formed the basis of the bioreactor, which was sterilized by autoclaving. Silicone tubing (sizes L/S 14 and L/S 16) was inserted through the silicone stopper to connect the lung. PA and PV perfusion loops allowed reseeding and constant media perfusion. The bioreactor was designed as a closed system that could be autoclaved after cleaning, to minimize the risk of contamination. For these experiments, the bioreactor had to be opened only twice: once at the time of organ loading, and once at organ culture day 1.

### Histology and immunohistochemistry

For paraffin-embedded tissue sections, samples were fixed in 4% paraformaldehyde in PBS for 2 h, processed for paraffin embedding, and cut into 5 μm sections. For H&E staining, sections were deparaffinized, rehydrated, stained with hematoxylin solution S (Merck) and eosin Y (Fischer Scientific), and mounted with Malinol (Muto Pure Chemical) after dehydration. Samples were imaged using a Provis AX-80 microscope (Olympus).

For immunofluorescence staining, samples were fixed in 4% paraformaldehyde in PBS for 2 h, processed for paraffin embedding, and cut into 5 μm sections. Then antigen retrieval was performed by incubating deparaffinized sections in 10 mM citrate buffer (pH 6) at 121 °C for 15 min. Sections were then blocked with 1% BSA/0.3% Triton X-100 in PBS for 1 h and incubated overnight in primary antibodies at 4 °C. Next, sections were washed three times with PBS and incubated in secondary antibodies for 1 h at room temperature followed by three washes in PBS. For ASC immunocytochemistry, cells in chamber slides were washed with PBS and fixed in methanol/acetone for 15 min at −20 °C. Slides were then blocked in PBS containing 1% BSA and incubated with either primary antibody overnight at 4 °C. Slides were washed three times with PBS and then incubated with secondary antibody for 1 h at room temperature followed by three washes in PBS. The primary antibodies used were anti-rat α-SMA (Sigma Aldrich), platelet endothelial cell adhesion molecule-1 (PECAM-1/CD31, Santa Cruz), NG-2 (Millipore), PDGFR-β (Cell Signaling Technologies), PCNA (Dako), collagen-IV (Abcam), and laminin (Abcam).

All stained sections were mounted in mounting medium containing DAPI (Vector Laboratories, Burlingame, CA) and imaged using a Leica DM6000 (Leica) or BZ-9000 BioRevo (Keyence) microscope.

### Scanning electron microscopy

Samples were fixed in 2% glutaraldehyde and 2.5% paraformaldehyde in 0.1 M cacodylate buffer (EMD Biosciences, Gibbstown, NJ) for 2 h at room temperature, then rinsed in cacodylate buffer, sliced, and dehydrated via an ethanol gradient. Samples were further dehydrated in hexamethyldisilazane for 10 min and dried overnight, then sputter-coated with gold and analyzed using a JOEL JXA-8600 scanning electron microscope.

### ASC isolation and culture

Inguinal adipose tissue was obtained from young adult male Fischer 344 rats (8–12 weeks old). ASCs were isolated from the adipose tissue according to Zuk *et al*.^49^, with minor modifications. Briefly, the washed adipose tissue was cut into small pieces and digested with collagenase (Celase, Cytori Therapeutics, Tokyo, Japan) in PBS for 30 min in a shaking water bath at 37 °C. The collagenase was subsequently inactivated with an equal volume of PBS/5% BSA. The mature adipocyte fraction was separated from the stromal vascular fraction by centrifugation (400 × g, 10 min) and the resulting cellular pellets were resuspended in adipose-derived stem cell growth medium (ADSC-GM, Lonza, Walkersville, MD). After successive filtration through 100- and 40-µm cell strainers, the freshly isolated cells were cultured in ADSC-GM at 37 °C. At day 2 after plating, all non-adherent cells were removed by washing and the medium was replaced. Primary cells were cultured for up to 10 days and upon reaching 90% confluence were defined as “Passage 0”, as previously described^50^. Passage-0 cells were used for all experiments, unless specified otherwise.

### EC culture

RLMVECs were purchased from VEC Technologies (Rensselaer, NY) and maintained in fibronectin-coated cell culture flasks (BD Biosciences) in microvascular EC growth medium (EGM-2MV, Lonza) with supplements (EGM-2MV Bullet Kit, Lonza). The culture medium was refreshed every 2 days. RLMVECs were passaged on reaching 80% confluence using 0.25% trypsin-EDTA (Nacalai Tesque, Kyoto, Japan) and were used for lentiviral transduction at passage 1.

### Lentiviral vector production and RLMVEC lentiviral infection

Viral particles were produced and used to infect RLMVECs. Lentiviral vectors were generated by lipofectamine-mediated virus infection of HEK 293T cells. HEK 293T cells (8 × 10^6^) were cultured in 3 mL of Opti-MEM and 5 mL of DMEM/10% fetal bovine serum (FBS) in 10-cm petri dishes and infected the following day. HEK cells were infected with 3 μg of transfer vector plasmid carrying the eGFP gene under the CMV promoter, 9 μg of package mix, and 36 μL of Lipofectamine 2000 (Thermo Fisher Scientific). The medium was exchanged for 10 mL DMEM/10% FBS 8 h after infection. Supernatant (10 mL) containing infectious particles was collected after 48 h. Viral supernatants were concentrated for preservation and were diluted (1:10) with EGM-2MV medium prior to infection of RLMVECs for 24 h. Three days after the initial viral infection, 10 μg/mL blasticidin S hydrochloride (Wako Pure Chemical, Osaka, Japan) was administered for 3 days to enrich the successfully labeled cells. The term eGFP-ECs refers to eGFP-RLMVECs in this study. Preservation of vascular phenotype and function in lentiviral vector-transfected RLMVECs was verified by flow cytometric analysis in comparison with untransfected RLMVECs (Supplementary Fig. 5).

### ASC and rat lung microvessel EC (RLMVEC) flow cytometric analysis

For flow cytometric analysis, cells were dissociated into single cell suspensions by incubation in trypsin/EDTA for 5 min. The dissociated cells were resuspended (1 × 10^5^ cells) in 100uL of FACS buffer (PBS + 5% FBS + 0.1% Sodium azide), and then incubated for 30 minutes at 4 °C. After washing twice with FACS buffer, cells were analyzed with a fluorescence-activated cell sorter (FACS) (FACS Canto II, Becton Dickinson, San Jose, CA), and the acquired data were analyzed (Cell Quest software, Becton Dickinson, San Jose, CA). Cultured ASCs were characterized using antibodies against rat CD73 (BD Biosciences, 551123), CD90-FITC (BD Biosciences, 554897), CD31-PE (BD Biosciences, 555027), CD34 (Santa Cruz Biotechnology, sc-7324), and CD45-FITC (BD Biosciences, 554877). Anti-CD34 and CD73 antibodies were labeled with Alexa Fluor 488 (Thermo Fisher Scientific) Species-specific fluorophore (Alexa-Fluor 488) conjugated secondary antibodies (Abcam).

Lentiviral vector-transfected RLMVECs were analyzed by FACS with mouse anti-rat CD90-FITC and CD31-PE to determine whether they retained their vascular phenotype (Supplementary Fig. 5).

### Differentiation of adipose-derived colony forming units

To induce osteogenesis, ASCs were cultured in MSC Osteogenic Differentiation Medium (Cyagen) for 4 weeks, and mineralized deposits were identified by Alizarin Red staining (Wako). Adipogenesis was induced by culturing ASCs in MSC Adipogenic Differentiation Medium (Cyagen) for 3 weeks, and Oil Red O staining was used to identify lipid-laden fat cells. Chondrogenic differentiation was assessed in cultures of ASCs grown in MSC Chondrogenic Differentiation Medium (Cyagen) for 3 weeks, and assessed by Alcian Blue staining for proteoglycan synthesis.

### Transduction of quantum dots (QDs) into ASCs

QDs are inorganic probes that consist of CdSe/ZnS-core/shell semiconductor nanocrystals. QD transduction with R8 (a transduction agent) can be used for labeling ASCs while maintaining stem cell potency with low cytotoxicity. QD labeling was performed here, as previously described^28^. Briefly, cultured ASCs were incubated with the R8-QDs655 complex (2 nM) in transduction medium (DMEM/F12, 2% FBS, 100 U/mL, penicillin/streptomycin) MSC culture medium at 37 °C for 4 h, followed by washing twice with the medium. After QDs655 transduction was confirmed by conventional fluorescence microscopy, the ASCs were reseeded into decellularized lungs on the same day. QD fluorescence was detected in formalin-fixed tissue specimens. ASC differentiation potential has been shown to be preserved after QDs655 labeling^46^.

### Rat lung scaffold re-endothelialization

Lung recellularization was performed using a previously published but modified protocol^34^. Immediately after trypsinization, 4 × 10^7^ eGFP-ECs, with and without 1 × 10^7^ QDs655-ASCs, were diluted in 100 mL of EGM-2MV medium. All cell-seeding experiments were conducted in the bioreactors, allowing cell delivery and perfusion from both the PA and PV. The PA and trachea were attached to the inside of the bioreactor via a fixed port. (Supplementary Fig. 4a,b). Decellularized lung scaffolds were primed by perfusion with 100 mL of PBS and equilibrated in the respective culture medium for at least 3 h before cell seeding. For re-endothelialization, *in vitro* expanded ECs and/or ASCs were resuspended in a single seeding chamber with 50 mL of EGM-2MV and 50 mL of ADSC-GM and seeded simultaneously by means of gravity perfusion through the PA and PV at a ratio of 1:1. Two-hour static culture was then performed to allow cell attachment, and cultue medium perfusion was initiated at 1 mL/min from both the PA and PV, and continued for 1 day after seeding. During vasculature perfusion, the airway branches were filled with EC media from the airway reservoir via tracheal cannulation to avoid exposure to air (Supplementary Fig. 4c,d). At day 1, the PV cannula was released, and perfusion was changed to 4 mL/min from the PA only until the end of the culture period. In the decellularized scaffold, we mixed FGF9 (R&D systems, 273-F9-025) with the culture medium (EGM-2MV + ADSC-GM) (50 ng/mL) at the time of static culture in the EC-ASC-FGF9 group, as previously described^27^. Media were refreshed every other day in all groups. Media were refreshed every other day. Regenerated lungs were then prepared for histological analysis, RNA extraction, frozen sectioning, raw sampling, and live tissue imaging, as described below.

### RNA extraction, reverse transcription, and quantitative PCR

Lung samples were incubated in RNA stabilization reagent for 24 h at 4 °C and stored at −80 °C. Total RNA isolation was performed with an RNAeasy Mini Kit (Qiagen) according to the manufacturer’s instructions. Reverse transcription and complementary DNA (cDNA) synthesis were performed using an RT^2^ First Strand Kit (Qiagen) and 1 μg RNA. Commercially available PCR arrays, including an Angiogenesis Array, were obtained from SA Biosciences (Frederick, MD). The PCR array contained 84 primer pairs that amplify genes involved in rat angiogenesis. Briefly, cDNA was mixed with 2 × RT^2^ SYBR Green Master Mix (1.35 mL; Qiagen) and RNase-free water to a final volume of 2.7 mL. Each well in the RT^2^ Profiler PCR array plate contained 25 μL of sample. PCR was performed on a Takara TP-800 thermal cycler (Takara, Shiga, Japan) following the manufacturer’s instructions. The housekeeping genes hypoxanthine phosphoribosyltransferase 1 (Hprt1) and ribosomal protein lateral stalk subunit P1 (Rplp1) were used to normalize expression levels, calculated using ΔCt values. Fold-changes in expression were calculated using the ΔΔCt (threshold cycle) method. ΔΔCt values in the EC and EC–ASC groups were determined and the fold-changes were calculated as 2^(−ΔΔCt)^.

### Quantification of pericyte-wrapped pulmonary vessels

For quantification of pericyte-wrapped pulmonary vessels, fluorescence images of CD31 and NG2 staining were taken in the same field of view using a Leica DM6000 microscope. The proportion of pulmonary vessels wrapped by NG2-positive mural cells (including partial wrapping) in the regenerated lungs at day 8 of culture was assessed in the EC, EC–ASC, and EC–ASC–FGF9 groups using four representative pulmonary section images (x20 magnification, n = 3 animals/group).

### Quantification of EC coverage

EC coverage was quantified as previously described^34^. Briefly, fluorescence images of CD31 and collagen IV were taken separately in the same field of view using a Keyence Biorevo BZ9000 microscope. Images were converted to binary images, skeletonized, and dilated using ImageJ software (NIH). Pixel numbers in the processed images were counted using ImageJ software, indicating EC coverage (CD31) or collagen IV coverage in the entire field. EC coverage of the regenerated lungs was normalized to collagen IV coverage. For each regenerated lung, pictures were taken from three representative fields at 20x magnification. Normalized EC coverage was quantified for the EC, EC–ASC, and EC–ASC–FGF9 groups at day 8 of culture (*n* = 3 animals/group) and presented relative to that of the native rat lung.

### EC survival assay

To determine EC viability in the regenerated lungs, GFP staining intensity per unit area was measured. GFP coverage was quantified for all groups on days 8 and 16 (*n* = 3 animals/group). EGFP channel images of equal magnification (40x) were captured for anti-GFP and DAPI immunofluorescence. Image analysis was performed using the ImageJ software. For threshold analysis, the images were digitally adjusted to remove background and increase the contrast. RGB images were converted to 8-bit images, and threshold determinations were used to digitally highlight all GFP-labeled cells. Finally, the percentage of highlighted pixels was calculated relative to the total tissue area of the field.

### TUNEL and PCNA assays

A Click-iT Plus TUNEL Assay for *In Situ* Apoptosis Detection using Alexa Fluor 594 dye (Thermo Fisher Scientific), and a PCNA assay (DAKO), were performed on paraffin-embedded sections according to the manufacturers’ protocols. After double staining with anti-CD31 antibody and either TUNEL or PCNA to detect apoptotic or proliferative ECs, respectively, the numbers of TUNEL- or PCNA-positive ECs was quantified from 40x magnification fields (n = 12 microscopic fields for TUNEL and PCNA assays, n = 3 animals/group).

### Live tissue imaging of regenerated lungs using multiphoton microscopy

Live tissue imaging of regenerated lungs was performed as previously described^51, 52^. Regenerated lungs were treated using sterile techniques and minced into small pieces (approximately 2–3 mm) using a scalpel. The tissue pieces were then washed and incubated with FITC-dextran (molecular weight 75 kDa and 10 kDa) and *Griffonia simplicifolia* isolectin IB4 conjugated with Alexa Fluor (Vector Laboratories) before observation. Nuclei were counterstained with Hoechst 33342 (Molecular Probes). A two-photon excitation laser microscope (TCS SP5 MP; Leica) was used to acquire images. Tissues were excited using multi-color laser lines, and emissions were collected through appropriate narrow bandpass filters. Each image was produced from an average of eight frames, after which the acquired images were processed to produce a 3D model.

### Orthotopic transplantation of regenerated lungs

To investigate the function of re-endothelialized pulmonary vasculature *in vivo*, orthotopic left lung transplantation was performed using the cuff technique as previously described^53^. Briefly, the regenerated lungs were isolated, and cuffs were placed on the PA, PV, and bronchus. Subsequently, the recipient rats were anesthetized with an intraperitoneal injection of ketamine/xylazine after inhalation of isoflurane in a glass chamber. After intubation, rats were connected to small animal ventilator (volume-cycled ventilator SN-480-7; Shinano Seisakusyo, Tokyo, Japan) and mechanically ventilated. Anesthesia was maintained with inhaled isoflurane. A left-sided thoracotomy in the fifth intercostal space was performed, and the recipient’s left lung was gently retracted using a binder clip, to expose the left hilum. The left PA, PV, and bronchus of the recipient were then dissected free of adjacent tissue. The cuffed PA, PV, and bronchus of the regenerated lung were inserted into the corresponding recipient structures, and the anastomoses were secured with 7-0 polypropylene. The recipient’s left lung was then removed following implantation of the regenerated lung. Finally, the incision was closed in layers and the animal was extubated. The regenerated lungs were left implanted for up to 3 h, after which the recipient animals were euthanized and the implanted lungs harvested.

### Statistical analysis

Numerical values are presented as the means ± standard deviation^54^. Because the sample sizes were too small to assess normality of distribution (N = 3), both parametric and non-parametric tests were performed. Statistical significance between the groups was evaluated using an unpaired Student’s t-test and a Mann–Whitney U test. Differences between more than two groups were assessed with ANOVA followed by a Bonferroni test, and a Kruskal–Wallis test followed by a Steel–Dwass test. All statistical analyses were performed using SPSS software. Values of *P* < 0.05 were considered significant.

## Electronic supplementary material

Supplementaly information
