# Supplementary material for: Transplantation of bioengineered rat lungs recellularized with endothelial and adipose-derived stromal cells
Source: Sci Rep. 2017 Aug 16;7:8447. doi: 10.1038/s41598-017-09115-2 (PMC5559597; doi:10.1038/s41598-017-09115-2)
Supplement: Supplementary file 1 — Supplementaly information [file 41598_2017_9115_MOESM1_ESM.pdf]

Transplantation of bioengineered lungs recellularized with endothelial and adipose-derived stromal cells

Ryoichiro Doi[1], Tomoshi Tsuchiya[1,2,*], Norisato Mitsutake[3], Satoshi Nishimura[4,5], Mutsumi Matsuu-Matsuyama[6], Yuka Nakazawa[7], Tomoo Ogi[8], Sadanori Akita[9], Hiroshi Yukawa[10], Yoshinobu Baba[10], Naoya Yamasaki[1,11], Keitaro Matsumoto[1,11], Takuro Miyazaki[1], Ryotaro Kamohara[1], Go Hatachi[1], Hideyori Sengyoku[1], Hironosuke Watanabe[1], Tomohiro Obata[1], Laura E. Niklason[12,13], and Takeshi Nagayasu[1,11,*]

[1] Department of Surgical Oncology, Nagasaki University Graduate School of Biomedical Sciences, Nagasaki 852-8501, Japan
[2] Translational Research Center, Research Institute for Science & Technology, Tokyo University of Science, Chiba 278-8510, Japan
[3] Department of Radiation Medical Sciences, Atomic Bomb Disease Institute, Nagasaki University, Nagasaki 852-8523, Japan
[4] Department of Cardiovascular Medicine, Translational Systems Biology and Medicine Initiative, Graduate School of Medicine, The University of Tokyo, Tokyo 113-8654, Japan
[5] Center for Molecular Medicine, Jichi Medical University, Tochigi 329-0498, Japan
[6] Department of Tumor and Diagnostic Pathology, Atomic Bomb Disease Institute, Nagasaki University, Nagasaki 852-8523, Japan
[7] Department of Genome Repair, Atomic Bomb Disease Institute, Nagasaki University, Nagasaki 852-8523, Japan
[8] Department of Genetics, Research Institute of Environmental Medicine, Nagoya University, Nagoya 464-8601, Japan
[9] Department of Plastic and Reconstructive Surgery, Nagasaki University Graduate School of Biomedical Sciences
[10] FIRST Research Center for Innovative Nanobiodevices, Graduate School of Engineering, Nagoya University, Nagoya 464-8603, Japan
[11] Medical-Engineering Hybrid Professional Development Center, Nagasaki University Graduate School of Biomedical Sciences
[12] Department of Biomedical Engineering, Yale University, New Haven, CT 06520, USA
[13] Department of Anesthesia, Yale University, New Haven, CT 06520, USA

*Corresponding authors: Tomoshi Tsuchiya, MD, PhD [Email: tomoshi@nagasaki-u.ac.jp], Takeshi Nagayasu, MD, PhD [Email: nagayasu@nagasaki-u.ac.jp]

Supplementary Figures

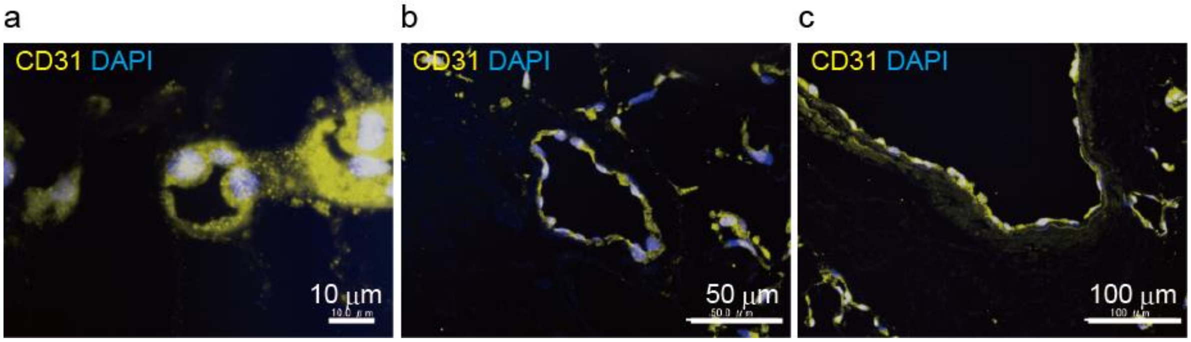

Supplementary Figure 1 | **Recellularized capillary, microvessel, and macrovessel of regenerated rat lungs forming a complete endothelium monolayer.** Immunofluorescence micrographs of thin sections from endothelial cell (EC)-regenerated lungs showing expression of CD31 (yellow) in the capillary (a), microvessels (b) and macrovessels (c). Nuclear DAPI (blue). A complete endothelium monolayer was formed.

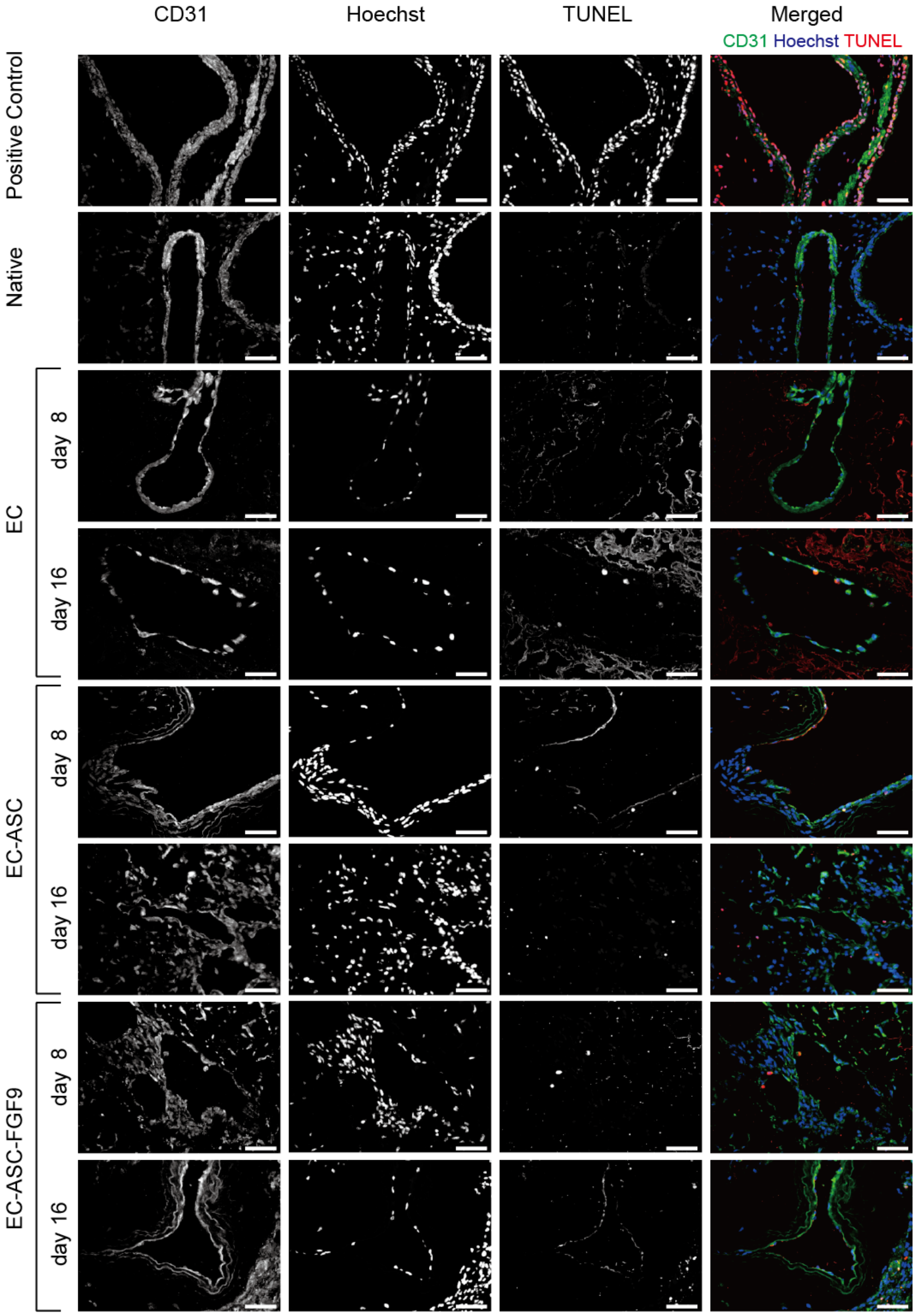

Supplementary Figure 2 | **Terminal deoxynucleotidyl transferase dUTP nick end labelling (TUNEL) staining of regenerated lungs.** Immunofluorescence micrographs of thin sections from native and EC-, EC-adipose-derived stem/stromal cell (ASC)-, and EC-ASC-fibroblast growth factor 9 (FGF9)-regenerated lungs showing TUNEL staining (red) and CD31 expression (green). Hoechst (blue). Scale bars: 100 µm.

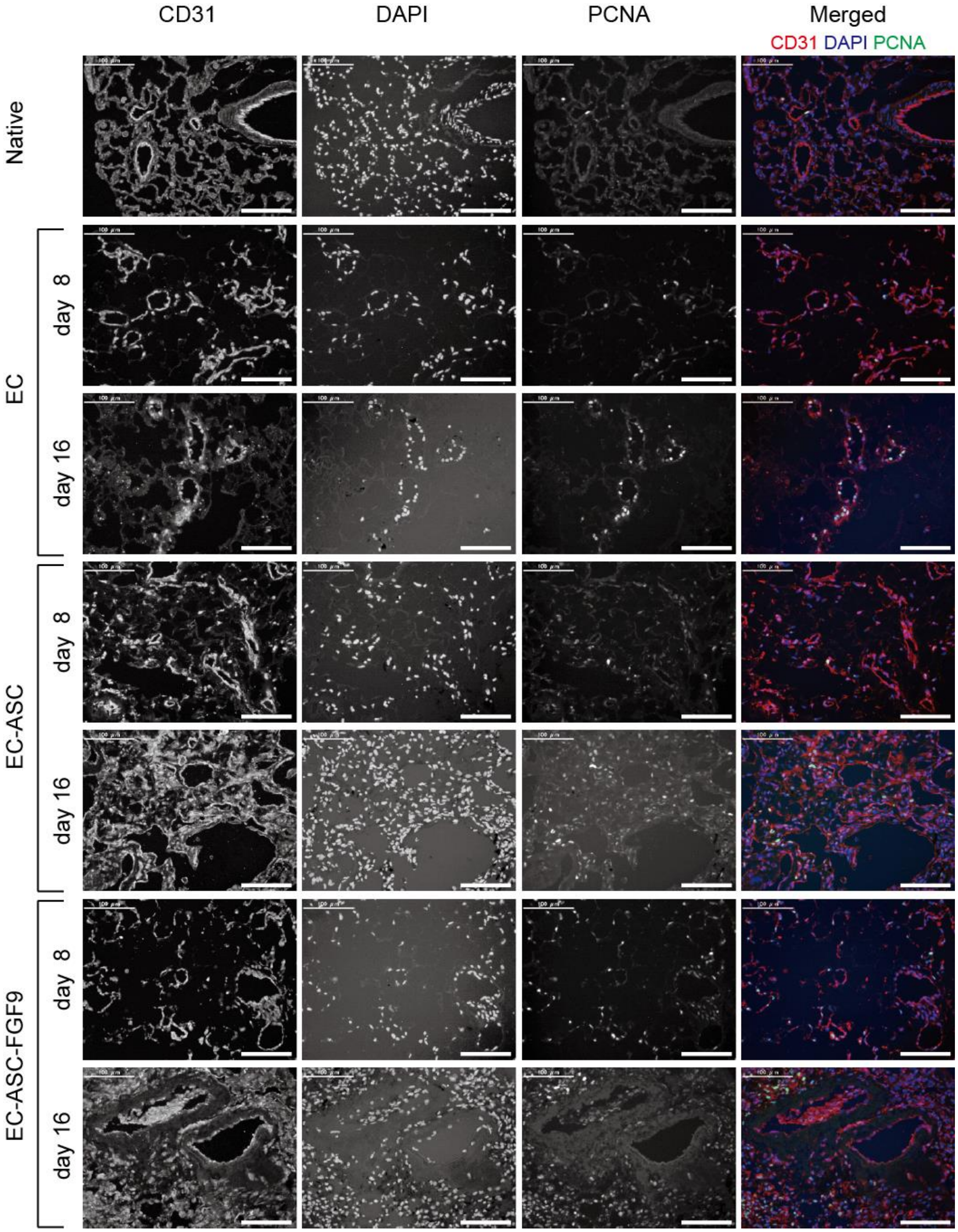

Supplementary Figure 3 | **Proliferating cell nuclear antigen (PCNA) assay of regenerated lungs.** Immunofluorescence micrographs of thin sections from native and EC-, EC-ASC-, and EC-ASC-FGF9-regenerated lungs showing expression of PCNA (green) and CD31 (red). Nuclear DAPI (blue) Scale bars: 100 µm.

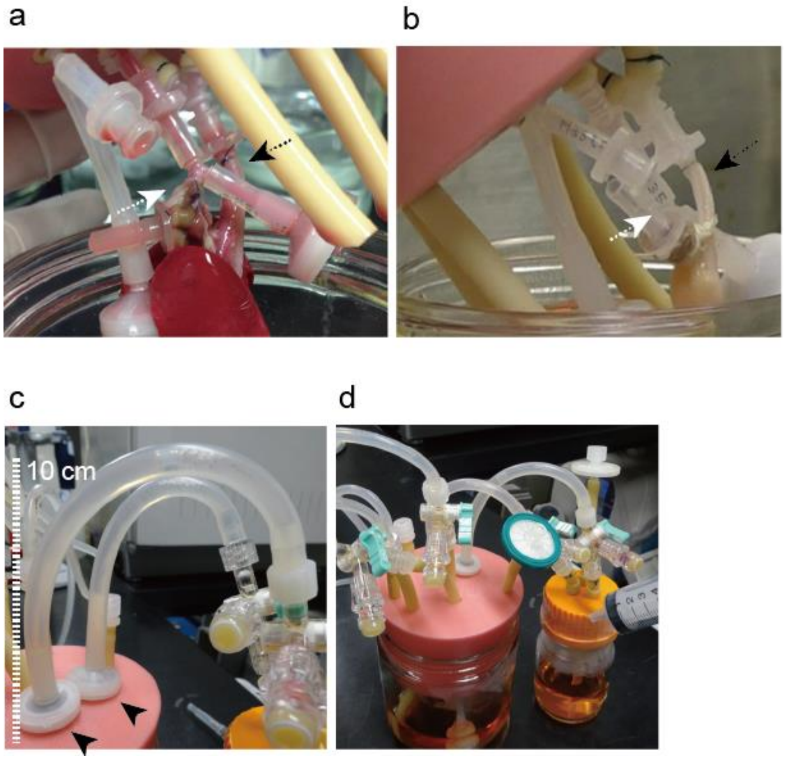

Supplementary Figure 4 | **Bioreactor system for recellularization.** The pulmonary artery (white arrow) and trachea (black arrow) were cannulated to the inside of the bioreactor through a fixed port (a, b). During vasculature perfusion, the airway branches were filled with EC media from the airway reservoir via trachea cannulation to avoid exposure to air (c, d).

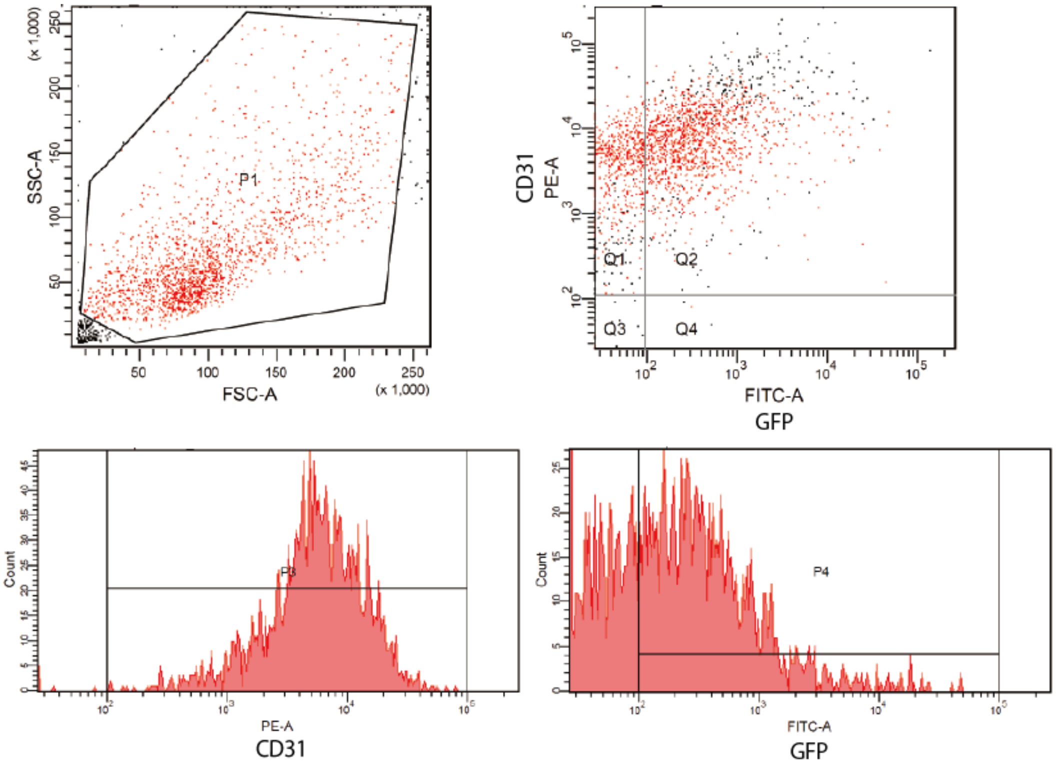

Tube: 4-RLMVEC with GFP CD31

Population	#Events	%Parent	%Total
All Events	2,441		100.0
P1	2,000	81.9	81.9
P3	1,993	99.6	81.6
P4	1,260	63.0	51.6
Q1	716	35.8	29.3
Q2	1,275	63.7	52.2
Q3	8	0.4	0.3
Q4	1	0.0	0.0

Supplementary Figure 5 | **Lentiviral vector-transfected GFP-rat lung microvessel ECs (RLMVECs) retained their vascular phenotype**. GFP-RLMVECs expressed the endothelial cell marker CD31.

Supplementary Table 1 | **Angiogeneis-related gene PCR array of the regenerated lungs**

Up-regurated Genes

Refseq	Symbol	Description	Fold Change	95% CI	P-value
NM_031012	Anpep	Alanyl (membrane) aminopeptidase	839.3	(0.00001, 2124.11)	0.0168
NM_053546	Angpt1	Angiopoietin 1	18.8	(0.00001, 65.44)	0.0290
NM_031761	Figf	C-fos induced growth factor	63.5	(0.00001, 189.51)	0.0212
NM_017017	Hgf	Hepatocyte growth factor	7.5	(3.10, 11.93)	0.0170
NM_001106549	Itgav	Integrin, alpha V	3.2	(1.07, 5.37)	0.0099
NM_031056	Mmp14	Matrix metallopeptidase 14 (membrane-inserted)	17.2	(0.00001, 44.50)	0.0085
NM_031054	Mmp2	Matrix metallopeptidase 2	7016.4	(4851.37, 9181.45)	0.0004
NM_030859	Mdk	Midkine	10.5	(4.36, 16.75)	0.0001
NM_177927	Serpinf1	Serpin peptidase inhibitor, clade F (alpha-2 antiplasmin), member 1	1043.0	(316.14, 1769.86)	0.0168
NM_133386	Sphk1	Sphingosine kinase 1	71.8	(30.94, 112.72)	0.0059
NM_001013062	Thbs1	Thrombospondin 1	8.6	(2.51, 14.72)	0.0208
NM_021989	Timp2	TIMP metallopeptidase inhibitor 2	19.7	(2.63, 36.76)	0.0028
NM_031836	Vegfa	Vascular endothelial growth factor A	21.2	(1.38, 41.04)	0.0052

Down-regulated Genes

Refseq	Symbol	Description	Fold Change	95% CI	P-value
NM_031530	Ccl2	Chemokine (C-C motif) ligand 2	0.14	(0.08, 0.20)	0.0111
NM_001010968	Eng	Endoglin	0.26	(0.10, 0.41)	0.0048
NM_053599	Efna1	Ephrin A1	0.14	(0.04, 0.23)	0.0345
NM_012797	Id1	Inhibitor of DNA binding 1	0.29	(0.09, 0.48)	0.0409
NM_013062	Kdr	Kinase insert domain receptor	0.09	(0.06, 0.13)	0.0003
NM_012801	Pdgfa	Platelet-derived growth factor alpha polypeptide	0.22	(0.14, 0.30)	0.0038
NM_017043	Ptgs1	Prostaglandin-endoperoxide synthase 1	0.28	(0.11, 0.45)	0.0035
NM_001105737	Tek	TEK tyrosine kinase, endothelial	0.24	(0.10, 0.38)	0.0325
NM_031131	Tgfb2	Transforming growth factor, beta 2	0.27	(0.19, 0.35)	0.0025
NM_012675	Tnf	Tumor necrosis factor (TNF superfamily, member 2)	0.12	(0.04, 0.20)	0.0237
NM_053545	Tie1	Tyrosine kinase with immunoglobulin-like and EGF-like domains 1	0.22	(0.11, 0.34)	0.0182
